# Chelating Fabrics Prepared by an Organic Solvent-Free Process for Boron Removal from Water

**DOI:** 10.3390/polym13071163

**Published:** 2021-04-05

**Authors:** Hiroyuki Hoshina, Jinhua Chen, Haruyo Amada, Noriaki Seko

**Affiliations:** Department of Advanced Functional Materials Research, Takasaki Advanced Radiation Research Institute, Quantum Beam Science Research Directorate, National Institutes for Quantum and Radiological Science and Technology, 1233 Watanuki-machi, Takasaki, Gunma 370-1292, Japan; hoshina.hiroyuki@qst.go.jp (H.H.); amada.haruyo@qst.go.jp (H.A.); seko.noriaki@qst.go.jp (N.S.)

**Keywords:** chelating fabric, boron removal, nonwoven fabric, radiation, graft polymerization, *N*-methyl-*D*-glucamine (NMDG)

## Abstract

A chelating fabric was prepared by graft polymerization of glycidyl methacrylate (GMA) onto a nonwoven fabric, followed by attachment reaction of N-methyl-D-glucamine (NMDG) using an organic solvent-free process. The graft polymerization was performed by immersing the gamma-ray pre-irradiated fabric into the GMA emulsion, while the attachment reaction was carried out by immersing the grafted fabric in the NMDG aqueous solution. The chelating capacity of the chelating fabric prepared by reaction in the NMDG aqueous solution without any additives reached 1.74 mmol/g, which further increased to above 2.0 mmol/g when surfactant and acid catalyst were added in the solution. The boron chelation of the chelating fabric was evaluated in a batch mode. Fourier transform infrared spectrophotometer (FTIR) was used to characterize the fabrics. The chelating fabric can quickly chelate boron from water to form a boron ester, and a high boron chelating ability close to 18.3 mg/g was achieved in the concentrated boron solution. The chelated boron can be eluted completely by HCl solution. The regeneration and stability of the chelating fabric were tested by 10 cycles of the chelation-elution operations. Considering the organic solvent-free preparation process and the high boron chelating performance, the chelating fabric is promising for the boron removal from water.

## 1. Introduction

**Scheme 1:**
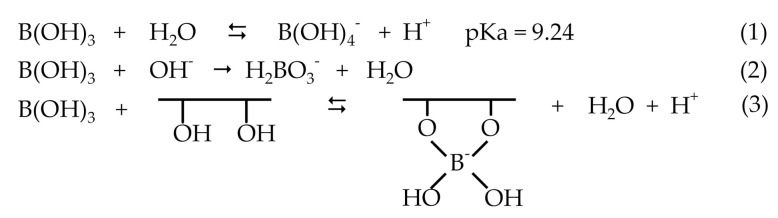
Possible reactions of boric acid in water.

The boron in seawater is about 4.5 mg/L, while in rivers it ranges from 0.3 to 100 mg/L. In Japan, the boron concentration in some hot-spring water even exceeds 100 mg/L. In natural water, it exists in the form of undissociated boric acid, which is a weak acid as shown in Scheme 1. The pKa of boric acid at room temperature is 9.24. Boric acid in natural water comes from the leaching of soils and rocks, as well as the wastewater from the glass/ceramic and metallurgy industries [1]. In an alkali solution it forms a borate salt, while in vicinal diol compound solution it forms boron ester.

The boron in water is of special concern because of its possible toxic effects on humans, animals, and plants. According to the WHO guidelines in 2011, boron in drinking water must not exceed 2.4 mg/L. Considering its impact on crops, boron in irrigation water is strictly regulated by many countries to be less than 0.5 mg/L [2,3,4].

The low-cost removal of boron from water has attracted considerable scientific interest [5,6]. Chemical precipitation is not suitable for removing trace boron from water. Ion-exchange resin is ineffective because boric acid exists as an unassociated compound in water. Membrane separation technologies, such as reverse osmosis membranes, are inadequate for rejecting boron because of the small size and neutral properties of the boric acid [7,8]. Adsorption methods using activated carbon, metal oxides, and other adsorbents for boron removal are also inefficient because of their low boron-adsorption [9].

Recently, chelating resins having vicinal diol groups, such as Amberlite^®^ IRA743 and DIAION^®^ CRB05, have been specifically designed for boron removal [10]. These chelating resins are composed of polystyrenic microporous frameworks and *N*-methyl-*D*-glucamine (NMDG) moieties. The boron removal is based on the chelation reaction shown in Scheme 1, Equation (3), where boric acid is chelated by the vicinal diol to form a stable five- or six-membered ring boron ester and release a proton [11,12,13]. The other frameworks, such as poly(glycidyl methacrylate-co-methyl methacrylate) [3], poly(glycidyl methacrylate-co-trimethylolpropane trimethacrylate) [14], poly(chloromethyl styrene-co-divinylbenzene) [7,15], and cellulose [16], have also been attached with the NMDG moieties to prepare the chelating resins for boron removal. These chelating resins are in the form of beads or particles, and are suitable for use in the column mode. Their boron chelating ability is strongly affected by the chelating capacity of the resins [13,17], and is hardly affected by the pH in a wide region of 4.0–8.0 and ionic strength of the aqueous solution [15,18]. In an acidic medium with a pH lower than 2.0, the chelated boron can be eluted according to the reverse reaction shown in Scheme 1, Equation (3). However, owing to their high porosity and crosslinking, these chelating resins are fragile and should be transported and stored in a water-swollen state.

On the other hand, nonwoven fabrics are easy to manufacture and have been widely used in the industry. As a fibrous porous medium nonwoven fabric has a high specific surface area. The phenomenon of liquid or gas transport through it has been well studied [19]. The polymeric nonwoven fabric can be chemically modified and converted into functional materials for water treatments [20]. Herein, a chelating fabric with NMDG moieties was designed. The chelating fabric can be used in both batch and column modes and transported and stored in the dry state [21]. For this purpose, glycidyl methacrylate (GMA)-grafted fabric is used as a framework to attach the NMDG moieties. The grafted fabric was prepared by radiation grafting the GMA onto a polyethylene-coated polypropylene (PE/PP) nonwoven fabric. Radiation grafting is an effective method to modify existing materials in any form of fiber, film, and fabric, giving them new properties such as reactivity, hydrophilicity, and conductivity [22,23]. The PE/PP nonwoven fabric has many advantages, including low cost, high mechanical strength, and easy radiation grafting [23,24,25]. The GMA monomer has vinyl groups and active epoxy rings. The former gives it high graft polymerizability, while the latter gives it high reactivity to introduce various new functions [26,27,28,29,30].

The attachment of NMDG moieties onto the GMA-grafted fabric is based on the substitution reaction of amine with the epoxy ring. The reaction was carried out in a heterogeneous system, wherein NMDG is dissolved in the solution, and the GMA-grafted fabric is in the solid state. Therefore, the reaction is significantly affected by the diffusion of NMDG into the fabric and the steric structure of the reactants [31,32]. Acids and bases can accelerate the reaction or increase its selectivity [33]. Until now, the reaction of GMA-grafted fabric with NMDG was carried out in mixture solvents such as 1,4-dioxane and water [22]. The organic solvent 1,4-dioxane enhances the affinity of the reaction solution to the fabric, while water improves the solubility of NMDG in the solution. In this solution more than 80% of the epoxy rings can be converted to NMDG moieties [34,35,36]. However, the use of organic solvents is harmful to the environment and human health. In this study, we carried out the attachment reaction in NMDG aqueous solution without the use of organic solvents. This attachment reaction in an organic solvent-free process has rarely been reported. This may be due to the low affinity of the GMA-grafted fabric to the aqueous solution and the low reactivity. To address these concerns, we used surfactants and acid/base catalysts to accelerate the reaction. The boron chelation of the resulting chelating fabric was evaluated in batch mode in a boron aqueous solution.

## 2. Experimental

### 2.1. Materials

The PE/PP nonwoven fabric was obtained from Kurashiki Textile Manufacturing Co., Ltd., Kurashiki, Japan. The thickness of the fabric was about 0.5 mm, and the diameter of the fabric fiber was about 13 µm. The chemical reagents of GMA, NMDG (also known as meglumine or methylglucamine), and boric acid (B(OH)_3_ or H_3_BO_3_) were purchased from Fujifilm Wako Pure Chemical Corporation, Tokyo, Japan, and were used as-received. Three surfactants, polyoxyethylene sorbitan monolaurate (POSM, or Tween^®^ 20), sodium n-dodecyl sulfate (SDS), and n-octyl trimethylammonium bromide (OTBr), along with KOH and HCl solutions, methanol, and 1,4-dioxane, were purchased from Kanto Chemical Co., Inc., Tokyo, Japan. The resistivity of water used for the washing and preparation processes was higher than 18.0 MΩ cm. The concentrations of monomer and NMDG solutions were presented in the form of weight percentage, and the concentrations of the boron aqueous solutions were presented as mg/L.

### 2.2. Preparation of Grafted Fabric

**Scheme 2:**
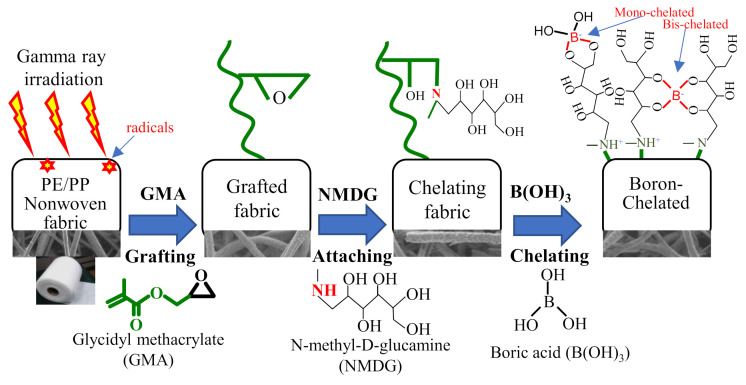
Preparation and boron chelation image of chelating fabric.

Scheme 2 shows the preparation process and the boron chelation image of the chelating fabric. For the preparation of GMA-grafted fabric, the PE/PP nonwoven fabric (14.06 g) was cut into 3.5 cm × 6.0 cm slices and placed into a three-necked reaction flask. The flask was vacuumed, filled with nitrogen, and then placed in a Co-60 irradiation room for gamma-ray irradiation. The oxygen was completely removed to avoid the generation of peroxide during irradiation. The irradiation dose rate was 1.5 kGy/h, and the total irradiation dose was 24 kGy. After irradiation, the flask was filled with GMA emulsion to completely immerse the fabric slices. The GMA emulsion was prepared by ultrasonic dispersion of a mixture of 40 g GMA, 4 g POSM surfactant, and 756 g water for 30 min before use. The flask was then placed in a bath at 40 °C for 4 h to initiate the graft polymerization. During grafting, nitrogen was bubbled into the emulsion at a rate of 5 mL/min to prevent oxygen from entering. After grafting, the fabric slices were thoroughly washed with water and methanol, and then dried in an oven at 60 °C for more than 24 h. Finally, a GMA-grafted fabric was obtained. The weight of the dry GMA-grafted fabric was 39.30 g, which corresponds to a degree of grafting of 179.5% and an epoxy density of 4.52 mmol/g.

### 2.3. Preparation of Chelating Fabric

For the preparation of chelating fabric, the GMA-grafted fabric was immersed into an NMDG solution at 80 °C to bring about a substitution reaction between the epoxy rings and NMDG molecules. The reaction was fixed at 80 °C and was investigated for up to 20 h. After reaction, the fabric was removed and washed thoroughly with methanol and water, and dried in an oven at 60 °C for 24 h. Thus, a chelating fabric with NMDG moieties was obtained. The conversion of epoxy rings to NMDG moieties and the chelating capacity (density of NMDG moieties) of the chelating fabric were calculated using the following equations.

Conversion (%) = (W_a_ − W_g_)/M_NMDG_/(D_e_ × W_g_/1000) × 100 (1)

Chelating capacity (mmol/g) = (W_a_ − W_g_)/M_NMDG_/(W_a_/1000) (2)

where W_a_ (mg) and W_g_ (mg) are the weights of the GMA-grafted fabric after and before the attachment reaction, respectively; M_NMDG_ (195.2 g/mol) is the molecular weight of NMDG; and D_e_ (4.52 mmol/g) is the epoxy density in the GMA-grafted fabric. We confirmed that the difference is very small for the chelating capacity under a same reaction condition. Therefore, only one experiment was performed for each condition to obtain the chelating capacity.

### 2.4. Characterization

Fourier transform infrared spectrophotometer (FTIR) in attenuated total reflectance (ATR) mode (Spectrum One, PerkinElmer, Inc., Tokyo, Japan) was used to analyze the fabric samples. The scanning range and resolution were 600–3800 cm^−1^ and 4.0 cm^−1^, respectively.

### 2.5. Evaluation of the Chelating Fabric

For the evaluation of the chelating fabric, the chelating fabric (about 100 mg) was immersed in a boron aqueous solution (35 mL) in a glass bottle and was shaken at a rate of 150 rpm at 25 °C, during which the boron was chelated by vicinal diol of the fabric. The chelating capacity of the chelating fabric is 2.15 mmol/g. From the viewpoint of practical use, boron aqueous solution was prepared by dissolving boric acid in deionized water without further adjusting the pH and ionic strength [10,30]. After chelation, the chelating fabric was immersed in 0.1 M HCl for boron elution. The chelating fabric was then washed thoroughly with 0.1 M KOH and water for regeneration.

The boron concentrations were detected by an ICP-OES system (Optima 8300, PerkinElmer, Inc., Tokyo, Japan). The samples were filtered through a 0.45 µm filter and then diluted with a 10-fold volume of 0.5% HNO_3_. The amount of boron chelation was calculated using the following equation.

Boron chelation (mg/g) = (C_0_ − C_i_) × V_a_/W_a_ (3)

where C_0_ and C_i_ are the boron concentrations (mg/mL) of the solution before and after chelation, respectively; V_a_ (mL) is the volume of the boron aqueous solution; and W_a_ (g) is the weight of the chelating fabric. It was found that the boron chelation detected in each experiment under the same conditions was slightly different. This may be due to the structure of the chelating fabric and the stirring effect. Even so, the error of the boron chelation was still less than 1.0%, and the chelating results showed a good and reasonable trend with the chelating time.

The reusability and stability of the chelating fabric were tested through 10 cycles of chelation-elution operations. This series of operations was performed under shaking at 25 °C. For each cycle, the chelating fabric is immersed in 30 mL boron solution (20.42 mg/L) for 30 min for chelation, immersed in 15 mL HCl solution (0.1 M) for 15 min for elution, and then water washed for 5 min, treated with 0.1 M KOH for 5 min, and water washed again thoroughly to regenerate the chelating fabric for the next cycle. The elution time was sufficient for the release of the chelated boron. We confirmed that more than 98% of the boron was eluted within 5 min.

To evaluate the stability, the chelating fabrics were immersed in water, 0.1 M HCl and 0.1 M KOH solutions, respectively, at 80 °C for 48 h. The weights of chelating fabrics before and after immersion in these solutions were weighed to evaluate their stability.

## 3. Results and Discussion

### 3.1. Preparation of GMA-Grafted Fabric

The GMA-grafted fabric was used as a framework for the attachment of NMDG moieties to prepare a chelating fabric. The GMA-grafted fabric was prepared by a preirradiation grafting technique [35].

In the first step of preparation, the starting fabric is irradiated with gamma rays. Under irradiation, some C-H and C-C covalent bonds split into free radicals. In the amorphous phase, the radicals can re-bond to form crosslinked structures or small molecules. However, in the crystal phase, they are quite stable and can survive for several days at room temperature [37]. When the irradiated fabric comes in contact with the GMA monomers, the radicals can initiate the graft polymerization.

Table 1 summarizes the grafting conditions and properties of the resulting GMA-grafted fabric. The high grafting of 179.5% and the corresponding epoxy density of 4.52 mmol/g can be expected to yield a fabric with high chelating capacity. When each epoxy ring is converted to one NMDG moiety, fabric with a high chelating capacity of 2.40 mmol/g can be obtained. The GMA-grafted fabric with a high grafting of 179.5% has sufficient strength in both wet and dry states. This can be attributed to the high mechanical strength of PP in the PE/PP fibers. A degree of grafting higher than 200% can be obtained easily by changing the reaction conditions. However, the stability of the resulting chelating fabric decreases due to the separation of the graft phase from the fibers. After grafting, the fabric becomes stiff and somewhat hydrophilic.

### 3.2. Preparation of Chelating Fabric

**Figure 1:**
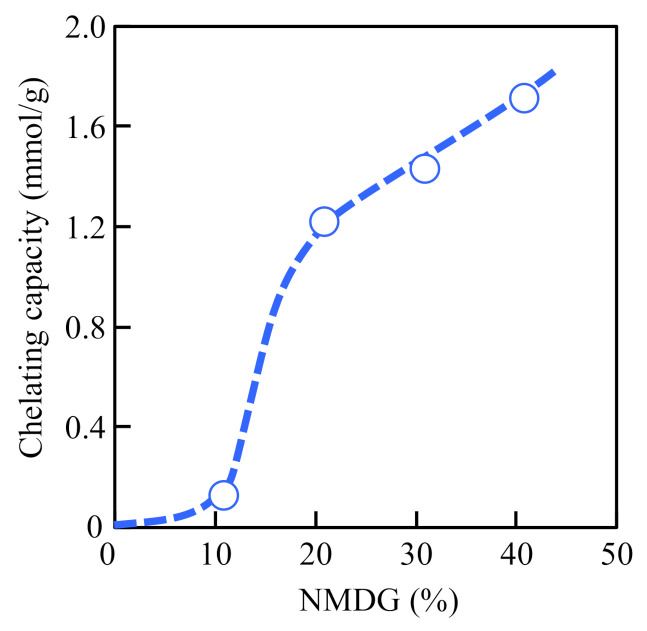
Effects of NMDG concentration in aqueous solution on chelating capacity of the resulting chelating fabrics. Reaction time, 3 h; temperature, 80 °C; without organic solvents.

The chelating fabric was prepared by immersing the GMA-grafted fabric in an NMDG aqueous solution with different concentrations up to 40%, which is close to the saturation concentration at room temperature. The reaction temperature was 80 °C and the reaction time was 3 h. No organic solvents were added to the reaction solution. Figure 1 shows the chelating capacity of the resulting fabrics as a function of the NMDG concentration. In the 40% NMDG solution, the chelating capacity reaches 1.74 mmol/g, which corresponds to 58.4% conversion of the epoxy rings to NMDG moieties. Because of steric hindrance and side reactions, a large number of epoxy rings are not converted to the NMDG moieties [38]. Prolonging the reaction time to 24 h or increasing the reaction temperature to 100 °C had almost no effect on the further increase in conversion. Nevertheless, considering the trade-off between performance and cost, the chelating fabric with a chelating capacity of 1.74 mmol/g is still acceptable for practical use.

In Figure 1, a threshold concentration appears between 10 and 20% concentrations. The chelating capacity is quite low at the 10% NMDG concentration and increased linearly above the 20% concentration. The low reactivity in the low NMDG concentration solution is because of the low affinity of the fabric toward the NMDG solution. When the GMA-grafted fabric was immersed into the 10% NMDG solution, initially, it was slightly white and was not completely wetted by the solution. In contrast, in the solution with an NMDG concentration of more than 20%, the GMA-grafted fabric was quickly wetted and completely swollen by the solution, indicating good affinity and reactivity between them. Increasing the NMDG concentration increases the possibility of contact between the NMDG molecules and epoxy rings, and decreases the polarity of the solution. This leads to an increased affinity between the solution and the fabric and thus increases the reactivity.

### 3.3. Enhancing the Attachment Reaction by Surfactants

**Figure 2:**
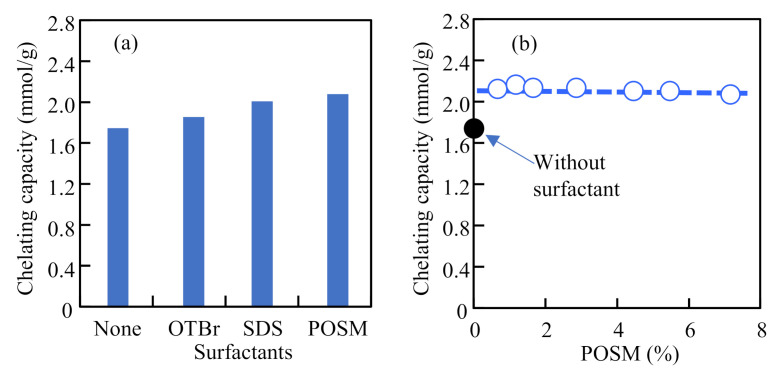
Effects of surfactants on the chelating capacity of the chelating fabric. NMDG aqueous solution, 40%; 80 °C; 3 h. (**a**) Effects of 1.0% surfactants of OTBr, SDS, and POSM. (**b**) Effects of POSM surfactant concentration.

We added surfactants to the NMDG solution to improve its affinity to the fabric. Three surfactants, cationic (OTBr), anionic (SDS), and non-ionic (POSM), were added at a concentration of 1.0% in 40% NMDG aqueous solutions. The reaction was controlled at 80 °C for 3 h. After adding the surfactants, the GMA-grafted fabric was fully wetted by the solutions quickly. As shown in Figure 2a, when there is no surfactant, the chelating capacity is 1.74 mmol/g, and it increases to 1.85, 2.00, and 2.08 mmol/g, with the addition of the surfactants of OTBr, SDS, and POSM, respectively. The increase in reactivity is caused by the improved affinity of the GMA-grafted fabric to the solution. The commercial Amberlite^®^ IRA743 chelating resin has an NMDG moiety density of 0.70 mmol/mL and a water content of about 50%, corresponding to a chelating capacity of about 1.9 mmol/g in the dry state [39]. The chelating capacity of 2.08 mmol/g of the resulting chelating fabric is close to that of the Amberlite^®^ IRA743 chelating resin.

Among the three surfactants, the addition of POSM resulted in the highest chelating capacity of 2.08 mmol/g. Furthermore, POSM has excellent properties such as low toxicity and good solubility in water. Figure 2b shows the effects of the POSM concentration on the chelating capacity. As expected, the chelating capacity is almost independent of the surfactant concentration. The critical micelle concentration (CMC) of POSM in water at room temperature is about 0.01%, which is much lower than that used in this study. Even a small amount of surfactant (less than 0.5%) can make the fabric well wetted by the solution. Therefore, the surfactant concentration used in this study is sufficient to enhance the affinity of the fabrics in solution [40].

### 3.4. Accelerating the Attachment Reaction by Base and Acid Catalysts

**Figure 3:**
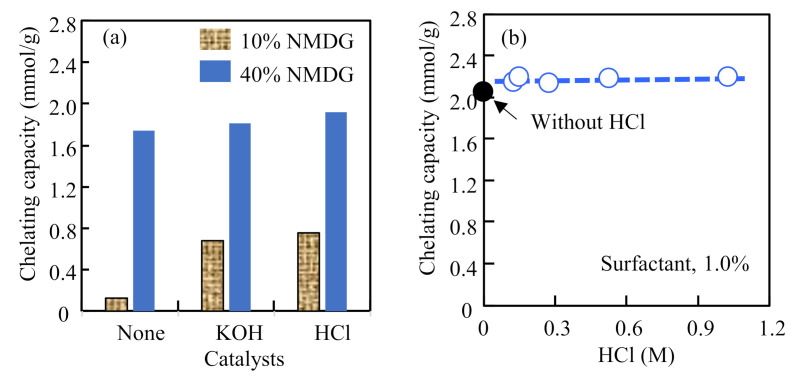
Effect of catalysts on chelating capacity of the chelating fabric. Reaction temperature, 80 °C; time, 3 h. (**a**) NMDG concentration, 10% and 40% in aqueous solution; catalyst, 0.1 M. (**b**) Effect of HCl concentration. NMDG concentration, 40%; POSM surfactant, 1.0%.

The attachment of NMDG onto the GMA-grafted fabric is an SN_2_ substitution reaction between the epoxy rings and secondary amine of NMDG [27,41,42]. Therefore, inorganic bases and acids can act as catalysts to accelerate the reaction. Figure 3a shows that, at a low concentration of 10% NMDG solution, the chelating capacity for the reaction without the catalyst is only 0.13 mmol/g, whereas by adding KOH and HCl it increases to 0.68 and 0.76 mmol/g, respectively. However, when a high concentration of 40% NMDG solution was used, the chelating capacity for the system without the catalyst was 1.74 mmol/g, and it slightly increased to 1.81 and 1.92 mmol/g, respectively, when KOH and HCl were added. Thus, at high NMDG concentrations, the catalyst effects on the reaction are relatively low.

HCl is a more effective catalyst than KOH. Figure 3b shows the effect of the HCl concentration on the chelating capacity. Here, 1.0% of the POSM surfactant was added to the reaction solution. As shown in Figure 3b, in the absence of HCl, the chelating capacity is 2.04 mmol/g. When HCl is added, it increases to 2.15–2.20 mmol/g and is almost independent of the HCl concentration. Thus, a low HCl concentration of 0.1 M is sufficient to catalyze the reaction.

**Scheme 3:**
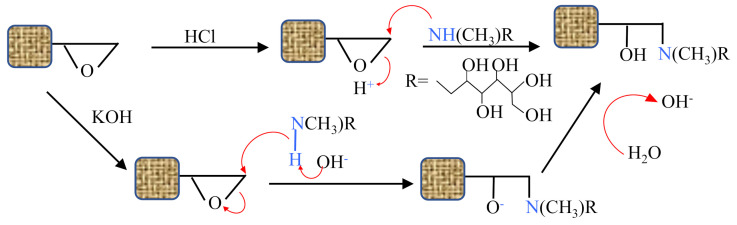
Substitution reaction of GMA-grafted fabric with NMDG catalyzed by HCl and KOH.

Scheme 3 shows the mechanism of reaction catalyzed by KOH and HCl. Owing to the strain of the three-membered rings, the epoxy rings on the GMA-grafted fabric are highly reactive. In the SN_2_ substitution reaction between the epoxy rings and NMDG, the secondary amine of NMDG attacks the carbon of epoxy rings and forms a new fabric with NMDG moieties [27,33]. The NMDG moiety is usually attached to the less hindered terminal carbon of the epoxy ring due to steric effects. When HCl is used as a catalyst, the oxygen of the epoxy ring is protonated by the acid, thereby increasing the reactivity of NMDG to attack the carbon of the epoxy ring. Conversely, when KOH is the catalyst, the secondary amine of NMDG is activated by the hydroxide ion, which is more conducive for attacking the carbon of epoxy ring [43,44]. Here, the GMA-grafted fabric is a solid material that is insoluble. The attachment reaction of NMDG onto the GMA-grafted fabric is a solid-phase reaction or a heterogeneous reaction. Compared with the general solution reaction, the reaction of the GMA-grafted fabric with NMDG is more complex, and the reaction rate and conversion are relatively low.

### 3.5. Reaction Kinetics

**Figure 4:**
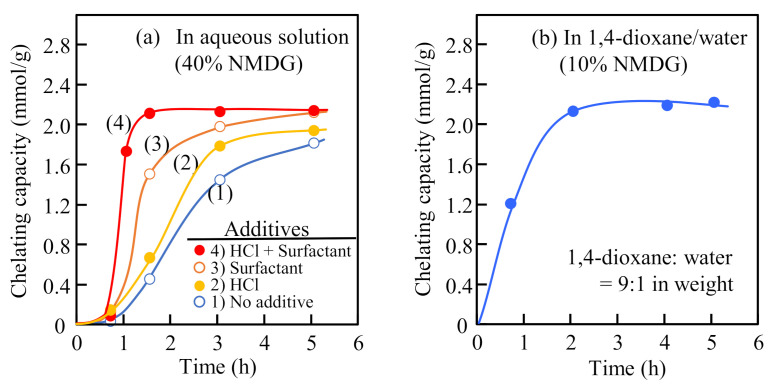
Kinetics of the reaction of GMA-grafted fabric in NMDG solution at 80 °C. (**a**) In 40% NMDG aqueous solution, and (**b**) in 10% NMDG 1,4-dioxane/water solution.

The reaction was confirmed to be enhanced by adding surfactants and catalysts to the reaction solutions. The reaction kinetics in the four conditions of the NMDG aqueous solution, (1) without any additives, (2) adding HCl as a catalyst, (3) adding POSM as a surfactant, and (4) adding both HCl and POSM, are summarized in Figure 4a. For comparison, the kinetics of the traditional attachment reaction in a 1,4-dioxane/water mixed solvent is shown in Figure 4b.

Figure 4a shows that there is an initial induction period during which the reaction is quite slow for all four conditions. This is because of the relative hydrophobicity and microporous structure of the fabric, which makes the NMDG diffuse slowly into the GMA-grafted fabric. After the fabric is well wetted by the solution, the NMDG molecules diffuse into the fabric and react with the epoxy rings quickly. The chelating capacity increased dramatically and reached a plateau level.

The rate of the reaction increased when the HCl catalyst and surfactant were added. Without any additives, the reaction plateaus after 5 h, whereas on adding both the catalyst and surfactant, the chelating capacity attains a high level of 2.0 mmol/g within 2 h. The surfactant improves the affinity of the fabric to the solution, and HCl catalyzes the substitution reaction by protonation of the oxygen atoms on the epoxy rings [41]. For comparison, the kinetics of the traditional reaction using a mixed solvent of 1,4-dioxane and water is shown in Figure 4b. The chelating capacity increases linearly with the increasing reaction time and reaches a value of 2.0 mmol/g within 2 h. No induction period was observed for this system. Consequently, the reaction in the aqueous solution containing the catalyst and surfactant is comparable to that in the solution with an organic solvent [36].

### 3.6. Boron Chelation in Aqueous Solution

**Figure 5:**
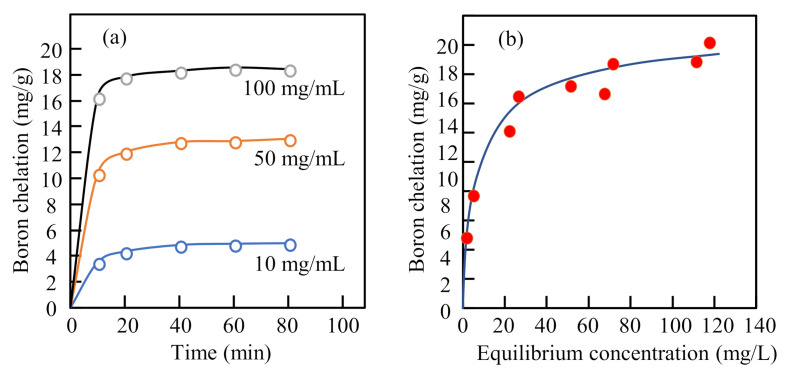
(**a**) Boron chelation kinetics of the chelating fabric in the initial concentrations of 10, 50, and 100 mg/L boron aqueous solution. (**b**) Relationship between boron chelation and equilibrium boron concentration in the remaining solution. Chelating time, 24 h; temperature, 25 °C.

Figure 5a shows the kinetics of boron chelation of the chelating fabric in boron aqueous solutions with the initial concentration of 10, 50, and 100 mg/mL. The chelating fabric with high chelating capacity of 2.15 mmol/g was used. The boron chelation is very fast, reaching a steady-state plateau within 20 min. The higher the initial boron concentration of the aqueous solution, the higher is the boron chelating ability obtained by the chelating fabric. The boron chelation of the chelating fabric reaches 17.8 mg/g in the 100 mg/L solution, 12.0 mg/g in the 50 mg/L solution, and further reduced to 4.3 mg/g in the 10 mg/L solution. In the 10 mg/L solution, all the boron is captured by the chelating fabric, while in the 100 mg/L solution, the chelating fabric is saturated to bond with boron. The chelating kinetics of boron chelation is similar to the results reported by Ting et al., where the attachment reaction was performed in a 10% 1,4-dioxane solution [45]. Therefore, the preparation of the chelating fabric without organic solvent is more environmentally friendly, and the resulted chelating fabric can be used to remove trace boron in drinking and irrigation water and effectively reduce boron concentration in wastewater.

Figure 5b shows the relationship between the boron chelation of the chelating fabric and the boron concentration of the remaining solution at 25 °C, recorded at a chelation time of 24 h, which is sufficient to establish an equilibrium state. For a low equilibrium of boron chelation of 4.3 mg/g, the equilibrium boron concentration in the remaining solution is too low to be accurately detected, indicating that the chelating fabrics can remove trace boron from water. In contrast, in the remaining solution with a boron concentration higher than 70 mg/L, a high boron chelating ability of 18.3 mg/g or 1.69 mmol/g is reached. Considering the NMDG density (2.15 mmol/g) of the chelating fabric, it can be concluded that about 50% of NMDG moieties were bonded with boron to form the mono-chelated structures and the remaining 50% were bonded in the form of bis-chelated structures (Scheme 2) [46,47].

### 3.7. FTIR Characterization

**Figure 6:**
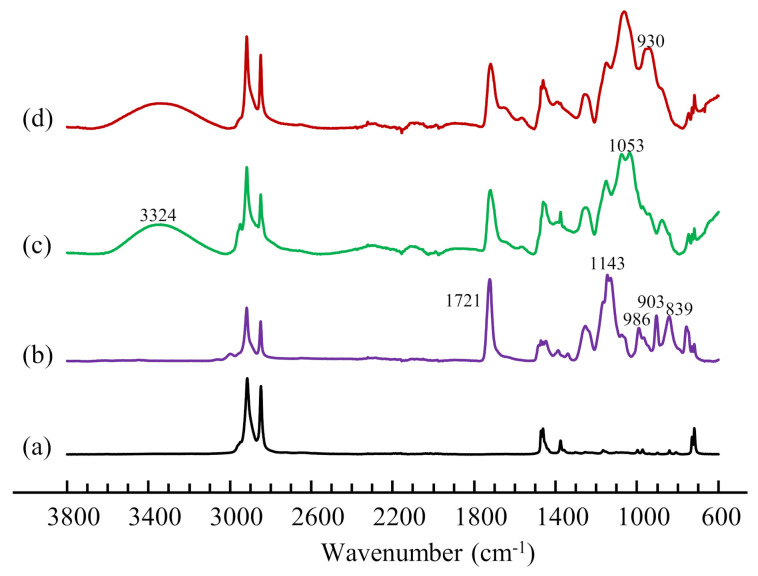
Fourier transform infrared spectrophotometer (FTIR) spectra of (**a**) nonwoven fabric, (**b**) GMA-grafted fabric, (**c**) chelating fabric prepared by attaching NMDG to GMA-grafted fabric, and (**d**) boron-chelated sample.

FTIR was used for characterization of the chelating fabrics before and after the boron chelation, the corresponding GMA-grafted fabric, and the starting nonwoven fabric. Figure 6 shows that the nonwoven fabric presents a simple spectrum because of the olefine chain of polyethylene. After grafting, the spectrum of the GMA-grafted fabric becomes complex and presents a strong peak around 1721 cm^−1^ assigned to the C=O structure, and three peaks at 986, 903, and 839 cm^−1^ assigned to the epoxy rings. After the attachment reaction with NMDG, very broad and strong peaks around 3324 cm^−1^ (assigned to the O-H bond) and 1053 cm^−1^ (assigned to C-O and C-N bonds) appear in the spectrum of the resulting chelating fabric. These results indicate that the GMA was grafted onto the fabric, and the chelating fabric was obtained by the attachment reaction of NMDG to the grafted fabric. The boron-chelated sample was obtained by immersing the chelating fabric (chelating capacity, 2.15 mmol/g) in concentrated boron aqueous solution for 30 min, followed by drying in oven at 60 °C for 24 h. A new peak around 930 cm^−1^, assigned to asymmetric B-O stretching, can be observed on the curve of boron-chelated sample (Figure 6d) [22,48].

### 3.8. Stability of the Chelating Fabric

**Figure 7:**
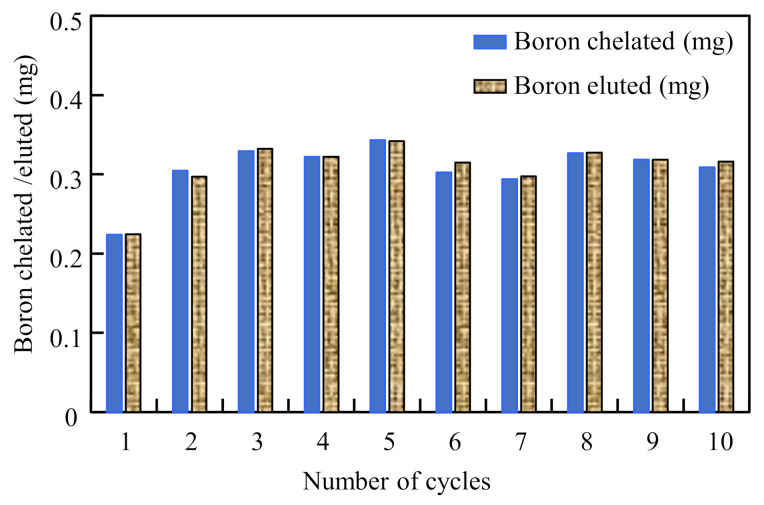
Stability of the chelating fabric under boron chelation-elution cycle test.

The chelating fabric is expected to be used repeatedly as a commercial chelating resin. Therefore, the chelating fabric should possess regeneration ability and high mechanical/chemical stability [49]. The regeneration of the chelating fabric includes HCl elution, KOH neutralization, and full water washing. As shown in Figure 7, 10 cycles of chelation-elution operations were performed, and the amount of boron chelated and eluted was determined.

As shown in Figure 7, the amount of the chelated boron on the new chelating fabric at the first cycle was 0.23 mg, increased to more than 0.31 mg after the second cycle, and then remained at a high level. The initial low chelating is due to the direct use of the dry chelating fabric without any pretreatment, where the boron diffusion is limited because the fabric is incompletely swollen. After the first cycle, the fabric is well wetted by the solution and the boron chelation is improved, resulting in a stable and high boron chelation.

In each chelation-elution cycle, the eluted boron is almost equal to that of the chelated boron. This indicates that the chelated boron can be completely eluted by the 0.1 M HCl acid. According to the reaction shown in Scheme 1, Equation (3), at a low proton concentration, the reaction of boric acid with vicinal diol tends to form boron ester, while at a high proton concentration, boric acid tends to get released. During the elution of boric acid, the amine on the chelating fabric reacts with HCl. Therefore, in addition to water washing, alkaline washing is needed to convert the amine [13].

After 10 cycles of chelation and elution, the chelating fabric was dried in an oven at 60 °C for 24 h. It was found that the weight of the chelating fabric dropped from 55.2 to 53.1 mg after the ten-cycle operations. This may be due to the strong shaking during the chelation-elution operations, which causes the fiber and the graft layer to separate from the fabric. Conversely, the chelating fabric was found to be very stable in water, 0.1 M HCl, and 0.1 M KOH solutions at 80 °C for 72 h, and there was no change in weight under the above treatments. Therefore, the chelating fabric with high boron-chelating ability can be durable in acid elution solution and alkali washing solution at high temperature, and can be reused to remove boron from water.

## 4. Conclusions

A chelating fabric was successfully prepared by radiation grafting GMA onto a nonwoven fabric, followed by an attachment reaction with NMDG using an organic solvent-free process. Addition of surfactant enhances the affinity of the fabric to the solution, while acid catalyzes the substitution reaction. Both the reaction rate and the conversion of epoxy rings to NMDG moieties of the reaction in aqueous solution are comparable to those utilizing organic solution.

The boron chelation of the resulting chelating fabric in water is very fast, reaching steady-state chelation within 20 min. The chelated boron can be quantitatively eluted with 0.1 M HCl solution within 5 min. The boron chelation reaches a high level of 18.3 mg/g in the concentrated boron aqueous solution. Furthermore, the chelating fabric is chemically stable in acid and alkali solutions, showing good durability and reusability for at least 10 cycles of chelation-elution operations. Therefore, it can be concluded that the chelating fabric is promising for boron removal from water. In future work, we will further optimize the process for the mass production of the chelating fabric, and investigate the cost feasibility of actual use in industry.

## Figures and Tables

**Table 1:** Grafting conditions and GMA-grafted fabric used in this study.

Grafting Conditions					Grafting Results	
Starting fabric	Preirradiation (kGy)	Monomer *	Temp. (°C)	Time (h)	Grafting (%)	Epoxy density (mmol/g)
PE/PP	24.0	GMA	40	4.0	179.5	4.52

* GMA monomer was dispersed in water at a concentration of 5.0% and emulsified by adding 0.5% POSM surfactant under ultrasonic treatment.

## Data Availability

Data is contained within the article.

## References

[1] Kochkodan V., Darwish N., Hilal N. (2015). The Chemistry of Boron in Water.

[2] Brdar-Jokanović M. (2020). Boron toxicity and deficiency in agricultural plants. Int. J. Mol. Sci..

[3] Wolska J., Bryjak M. (2013). Methods for boron removal from aqueous solutions—A review. Desalination.

[4] Hilal N., Kim G., Somerfield C. (2011). Boron removal from saline water, a comprehensive review. Desalination.

[5] Guan Z., Lv J., Bai P., Guo X. (2016). Boron removal from aqueous solutions by adsorption—A review. Desalination.

[6] Xu Y., Jiang J. (2008). Technologies for boron removal. Indus. Chem. Res..

[7] Samatya S., Tuncel S., Kabay N. (2015). Boron removal from RO permeate of geothermal water by monodisperse poly (vinylbenzyl chloride-co-divinylbenzene) beads containing N-methyl-D-glucamine. Desalination.

[8] Tu K., Long D., Allan R. (2010). Boron removal by reverse osmosis membranes in seawater desalination applications. Separ. Purif. Tech..

[9] Isaacs-Paez E., Leyva-Ramos R., Jacobo-Azuara A., Martinez-Rosales J., Flores-Cano J. (2014). Adsorption of boron on calcined AlMg layered double hydroxide from aqueous solutions. Mechanism and effect of operating conditions. Chem. Eng. J..

[10] Bıçak N., Özbelge H., Yilmaz L., Senkal B. (2000). Crosslinked polymer gels for boron extraction derived from N-glucidol-N-methyl-2-hydroxypropyl methacrylate. Macromol. Chem. Phys..

[11] Köse D.A., Zümreoglu-Karan B. (2009). Complexation of boric acid with vitamin C. New J. Chem..

[12] Manjunatha R., Gerbec J., Shimizu F., Chmelka B. (2019). Nanoscale surface compositions and structures influence boron adsorption properties of anion exchange resins. Langmuir.

[13] Kamcev J., Taylor M., Shin D., Jarenwattananon N., Colwell K., Long J. (2019). Functionalized porous aromatic frameworks as high-performance adsorbents for the rapid removal of boric acid from water. Adv. Mater..

[14] Wang L., Qi T., Gao Z., Zhang Y., Chu J. (2007). Synthesis of N-methylglucamine modified macroporous poly(GMA-co-TRIM) and its performance as a boron sorbent. React. Funct. Polym..

[15] Ting T., Nasef M., Hashim K. (2015). Tuning N-methyl-D-glucamine density in a new radiation grafted poly (vinyl benzyl chloride)/nylon-6 fibrous boron-selective adsorbent using the response surface method. RSC Adv..

[16] Wei Y.T., Zheng Y.M., Chen J.P. (2011). Functionalization of regenerated cellulose membrane via surface-initiated atom transfer radical polymerization for boron removal from aqueous solution. Langmuir.

[17] Kang J., Tang Y., Gao S., Liu L. (2018). One-dimensional controllable crosslinked polymers grafted with N-methyl-D-glucamine for effective boron adsorption. New J. Chem..

[18] Sabarudin A., Oshita K., Oshima M., Motomizu S. (2005). Synthesis of cross-linked chitosan possessing N-methyl-d-glucamine moiety (CCTS-NMDG) for adsorption/concentration of boron in water samples and its accurate measurement by ICP-MS and ICP-AES. Talanta.

[19] Xiao B., Zhang Y., Wang Y., Jiang G., Liang M., Chen X., Long G. (2019). A fractal model for Kozeny–Carman constant and dimensionless permeability of fibrous porous media with roughened surfaces. Fractals.

[20] Gu J., Xiao P., Chen P., Zhang L., Wang H., Dai L., Song L., Huang Y., Zhang J., Chen T. (2017). Functionalization of biodegradable PLA nonwoven fabric as superoleophilic and superhydrophobic material for efficient oil absorption and oil/water separation. ACS Appl. Mater. Interfaces.

[21] Recepoğlu Y., Kabay N., Ipek I., Arda M., Yüksel M., Yoshizuka K., Nishihama S. (2018). Packed bed column dynamic study for boron removal from geothermal brine by a chelating fiber and breakthrough curve analysis by using mathematical models. Desalination.

[22] Nallappan M., Nasef M., Ting T., Ahmad A. (2018). An optimized covalent Immobilization of glucamine on electrospun nanofibrous poly (vinylidene fluoride) sheets grafted with oxirane groups for higher boron adsorption. Fibers Polym..

[23] Nasef M., Güven O. (2012). Radiation-grafted copolymers for separation and purification purposes, Status, challenges and future directions. Prog. Polym. Sci..

[24] Hayashi N., Chen J., Seko N. (2018). Nitrogen-containing fabric adsorbents prepared by radiation grafting for removal of chromium from wastewater. Polymers.

[25] Kodama Y., Barsbay M., Güven O. (2014). Poly (2-hydroxyethyl methacrylate) (PHEMA) grafted polyethylene/polypropylene (PE/PP) nonwoven fabric by γ-initiation, Synthesis, characterization and benefits of RAFT mediation. Radia. Phys. Chem..

[26] Dong Z., Zhao L. (2018). Covalently bonded ionic liquid onto cellulose for fast adsorption and efficient separation of Cr (VI), Batch, column and mechanism investigation. Carbohyd. Polym..

[27] Muzammil E., Khan A., Stuparu M. (2017). Post-polymerization modification reactions of poly (glycidyl methacrylate)s. RSC Adv..

[28] Kimmins S., Wyman P., Cameron N. (2014). Amine-functionalization of glycidyl methacrylate-containing emulsion-templated porous polymers and immobilization of proteinase K for biocatalysis. Polymer.

[29] Chen J., Asano M., Yamaki T., Yoshida M. (2006). Preparation and characterization of chemically stable polymer electrolyte membranes by radiation-induced graft copolymerization of four monomers into ETFE films. J. Membr. Sci..

[30] Darwish N.B., Kochkodan V., Hilal N. (2017). Microfiltration of micro-sized suspensions of boron-selective resin with PVDF membranes. Desalination.

[31] Ehlers J., Rondan N., Huynh L., Pham H., Marks M., Truong T. (2007). Theoretical study on mechanisms of the epoxy− amine curing reaction. Macromolecules.

[32] Jouyandeh M., Shabanian M., Khaleghi M., Paran S., Ghiyasi S., Vahabi H., Formela K., Puglia D., Saeb M. (2018). Acid-aided epoxy-amine curing reaction as reflected in epoxy/Fe_3_O_4_ nanocomposites, Chemistry, mechanism, and fracture behavior. Prog. Org. Coat..

[33] Mijovic J., Fishbain A., Wijaya J. (1992). Mechanistic modeling of epoxy-amine kinetics. 1. Model compound study. Macromolecules.

[34] Ikeda K., Umeno D., Saito K., Koide F., Miyata E., Sugo T. (2011). Removal of boron using nylon-based chelating fibers. Indus. Eng. Chem. Res..

[35] Seko N., Ninh N., Tamada M. (2010). Emulsion grafting of glycidyl methacrylate onto polyethylene fiber. Rad. Phys. Chem..

[36] Hoshina H., Seko N., Ueki Y., Tamada M. (2007). Synthesis of graft adsorbent with N-methyl-D-glucamine for boron adsorption. J. Ion Exch..

[37] Forster A., Tsinas Z., Al-sheikhly M. (2019). Effect of irradiation and detection of long-lived polyenyl radicals in highly crystalline ultra-high molar mass polyethylene (UHMMPE) fibers. Polymers.

[38] Mora A.S., Tayouo R., Boutevin B., David G., Caillol S. (2020). A perspective approach on the amine reactivity and the hydrogen bonds effect on epoxy-amine systems. Eur. Polym. J..

[39] Mun D., Huynh N., Shin S., Kim Y., Kim S., Shul Y., Cho J. (2017). Facile isomerization of glucose into fructose using anion-exchange resins in organic solvents and application to direct conversion of glucose into furan compounds. Res. Chem. Intermed..

[40] Sar P., Ghosh A., Scarso A., Saha B. (2019). Surfactant for better tomorrow, applied aspect of surfactant aggregates from laboratory to industry. Res. Chem. Intermed..

[41] Eddingsaas N., Vandervelde D., Wennberg P. (2010). Kinetics and products of the acid-catalyzed ring-opening of atmospherically relevant butyl epoxy alcohols. J. Phys. Chem. A.

[42] Stropoli S., Elrod M. (2015). Assessing the potential for the reactions of epoxides with amines on secondary organic aerosol particles. J. Phys. Chem. A.

[43] Innocenzi P., Kidchob T., Yoko T. (2005). Hybrid organic-inorganic sol-gel materials based on epoxy-amine systems. J. Sol-Gel Sci. Techn..

[44] Blank W., He Z., Picci M. (2002). Catalysis of the epoxy-carboxyl reaction. J. Coat. Tech..

[45] Ting T., Nasef M. (2017). Modification of polyethylene-polypropylene fibers by emulsion and solvent radiation grafting systems for boron removal. Fibers. Polym..

[46] Joshi M., Chalumot G., Kim Y., Anderson J. (2012). Synthesis of glucaminium-based ionic liquids and their application in the removal of boron from water. Chem. Commun..

[47] Joshi M., Steyer D., Anderson J. (2012). Evaluating the complexation behavior and regeneration of boron selective glucaminium-based ionic liquids when used as extraction solvents. Anal. Chim. Acta.

[48] Akan Z., Demiroglu H., Avcibasi U., Oto G., Ozdemir H., Deniz S., Basak A.S. (2014). Complexion of Boric Acid with 2-Deoxy-D-glucose (DG) as a novel boron carrier for BNCT. Med. Sci. Discov..

[49] Wang B., Guo X., Bai P. (2014). Removal technology of boron dissolved in aqueous solutions–a review. Coll. Surf. A Phys. Eng. Asp..

